# Effects of Air Purifiers on the Spread of Simulated Respiratory Droplet Nuclei and Virus Aggregates

**DOI:** 10.3390/ijerph18168426

**Published:** 2021-08-10

**Authors:** Ki Joon Heo, Inyong Park, Gunhee Lee, Keejung Hong, Bangwoo Han, Jae Hee Jung, Sang Bok Kim

**Affiliations:** 1 Department of Environmental Machinery, Korea Institute of Machinery and Materials, Daejeon 34103, Korea; heorlwns@gmail.com (K.J.H.); ipark@kimm.re.kr (I.P.); gunhee@kimm.re.kr (G.L.); elsha@kimm.re.kr (K.H.); bhan@kimm.re.kr (B.H.); 2 Department of Mechanical Engineering, Sejong University, Seoul 05006, Korea

**Keywords:** air purifier, COVID-19, transmission, respiratory droplet nuclei, virus aggregates

## Abstract

The present study was performed to quantitatively evaluate the effects of air purifiers on the spread of COVID-19 and to suggest guidelines for their safe use. To simulate respiratory droplet nuclei and nano-sized virus aggregates, deionized water containing 100 nm of polystyrene latex (PSL) particles was sprayed using a vibrating mesh nebulizer, and the changes in the particle number concentration were measured for various locations of the particle source and air purifier in a standard 30 m^3^ test chamber. The spread of the simulated respiratory droplet nuclei by the air purifier was not significant, but the nano-sized aggregates were significantly affected by the airflow generated by the air purifier. However, due to the removal of the airborne particles by the HEPA filter contained in the air purifier, continuous operation of the air purifier reduced the number concentration of both the simulated respiratory droplet nuclei and nano-sized aggregates in comparison to the experiment without operation of the air purifier. The effect of the airflow generated by the air purifier on the spread of simulated respiratory droplet nuclei and nano-sized aggregates was negligible when the distance between the air purifier and the nebulizer exceeded 1 m.

## 1. Introduction

COVID-19 is still a worldwide threat, with more than 191 million confirmed cases and almost 4.1 million deaths as of 20 July 2021 [1]. Scientific investigations, political efforts, and public health guidelines have combined in an attempt to end the COVID-19 pandemic.

The spread of COVID-19 can be categorized into surface or fomite transmission, direct contact, and airborne transmission [2,3]. In the case of surface transmission, the virus can spread by direct contact with fomites. Viruses are known to remain infectious on contaminated objects for about 4–6 d [4]. To prevent surface transmission, frequent sanitization of public environments is highly recommended. The respiratory droplets, usually >5 μm, contain large amounts of the virus, and therefore, direct contact with respiratory droplets can infect susceptible hosts with a high probability. However, due to the large size of respiratory droplets, they sediment quickly within 2 m from infected individuals. Therefore, maintaining social distancing and wearing a mask can prevent the risk of infection by direct contact. Respiratory droplet nuclei < 5 μm in diameter can be transmitted through the air [2,3]. Recent studies showed that the airflow generated by ventilation facilities can increase the spread of respiratory droplet nuclei [5]. Therefore, care must be taken in the location of ventilation facility intake and exhaust. Furthermore, the relative humidity can also affect the spread of respiratory droplet nuclei [6]. Under conditions of low humidity, the moisture in the respiratory droplet nuclei can evaporate completely, and a single virus or aggregates of viruses, which can be nanometers in size, can be generated [6]. Although the inhaled dose of nano-sized virus aggregates is very low, they can be suspended and accumulate in the air for more than 12 h. Therefore, the probability of infection can increase continuously without removal of these nano-sized virus aggregates.

The most effective method to reduce the probability of infection is ventilation [7]. Natural and mechanical ventilation dilute the concentration of virus-bearing respiratory droplet nuclei and virus aggregates by supplying clean air and transporting the respiratory droplet nuclei and virus aggregates to the outside environment [8]. In addition to ventilation, air purifiers with high-efficiency particulate air (HEPA) filters are recommended by the US Environmental Protection Agency (EPA) to help remove suspended airborne particles that can contain viruses [9]. However, a recent pilot experiment showed that the exhaust airflow of an air purifier can spread airborne particles, particularly if the source of the droplets is placed near the exhaust fan of the air purifier [10]. As air purifiers can also remove airborne particles, questions remain regarding whether the use of an air purifier can reduce the possibility of infection or increase airborne transmission.

Here, we quantitatively evaluated the effects of an air purifier on the spread of COVID-19 using simulated respiratory droplet nuclei and nano-sized virus aggregates. The changes in the number concentration of simulated respiratory droplet nuclei and nano-sized virus aggregates in a standard test chamber were measured. To suggest guidelines for the safe use of air purifiers, we examined the spreading characteristics of the simulated respiratory droplet nuclei and nano-sized virus aggregates by varying the relative locations of the source of the simulated droplet nuclei and nano-sized aggregates and the air purifier.

## 2. Materials and Methods

A standard 30 m^3^ test chamber (W: 4 m × D: 3 m × H: 2.53 m) was used to evaluate the effects of the air purifier on the spread of saliva-like particles. The temperature and relative humidity were maintained at 26.6 ± 0.7 ℃ and 66.7 ± 5.5% in all experiments. A commercially available air purifier (AX34T3000WWD; Samsung Electronics, Suwon, Korea) was used for all experiments. The air purifier takes in air on one of its sidewalls and exhausts the purified air in the upward direction. The air purifier contains a filtration package consisting of three functional layers: a support layer with a pre-filter, absorption layers with granules of activated carbon, and a dust filtration layer with an H13 grade HEPA filter (CFX-G100D; 3AC Co., Ltd., Seoul, Korea). All experiments were performed with the air purifier in turbo mode to maximize the airflow, with an estimated flow rate of 219 m^3^/h.

**Figure 1:**
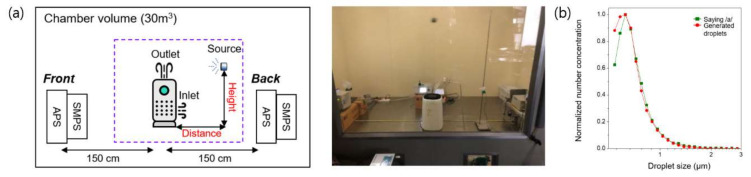
Experimental setup and the size distribution of the simulated respiratory droplet nuclei. (**a**) Schematic and photograph of the experimental setup; (**b**) comparison between the actual and simulated respiratory droplet nuclei.

To simulate the respiratory droplet nuclei generated by an infected individual, we suspended polystyrene latex (PSL; Duke Scientific Corp., Palo Alto, CA, USA) particles 100 nm in diameter in deionized water and sprayed the solution using a vibrating mesh nebulizer (HL100A; Health & Life Co., Ltd., New Taipei City, Taiwan). The size distribution and concentration of the generated simulated respiratory droplet nuclei were determined using an aerodynamic particle sizer (APS) (model 3321; TSI Inc., Shoreview, MN, USA), which is standard equipment for aerosol measurement, and can detect particles ranging in size from 0.5 μm to 20 μm. To measure the size distribution and concentration of the nano-sized aggregates, a scanning mobility particle sizer (SMPS) (Model 3080; TSI Inc., Shoreview, MN, USA), which can detect particles ranging in size from 10 nm to 300 nm, was used as shown in Figure 1a.

We evaluated the effects of the air purifier on the spread of COVID-19 by measuring the concentration of simulated respiratory droplet nuclei for various distances between the air purifier and the nebulizer, as well as heights of the nebulizer. To prevent the infiltration of particles from outside of the chamber, we sealed the chamber and confirmed the background noise level of the particle concentration before all experiments.

## 3. Results and Discussion

The size distribution of the simulated respiratory droplet nuclei was measured using an APS. To measure the actual respiratory droplet nuclei generated while speaking, one of the authors sounded /a/ at the inlet of the APS and determined the size distribution of saliva droplets. Figure 1b shows a comparison of the size distribution of the simulated respiratory droplet nuclei and actual salvia droplets. The size distributions were normalized by their peak number concentration. As shown in Figure 1b, the simulated respiratory droplet nuclei generated using the nebulizer had a similar size distribution to actual respiratory droplet nuclei. Most of the simulated and actual respiratory droplet nuclei were <1 μm in diameter and showed a peak at 0.54 μm. These results agreed well with a previous study [11].

**Figure 2:**
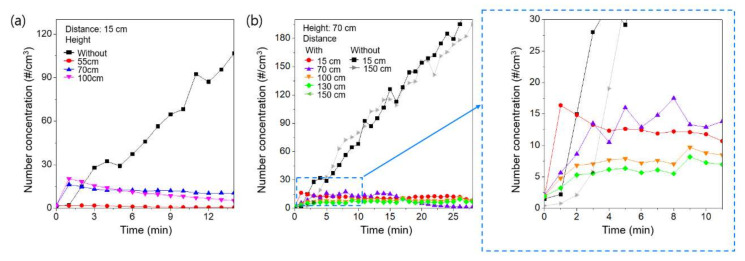
The change in the concentration of simulated respiratory droplet nuclei as a function of time (**a**) for various heights of the source and (**b**) for various distances between the air purifier and the source.

To evaluate the effects of the air purifier on the spread of simulated respiratory droplet nuclei, we followed pilot experiments performed previously by Ham [10]. We varied the height of the vibrating mesh nebulizer maintained at a constant distance of 15 cm from the air purifier. Figure 2a shows the changes in the concentration of the simulated respiratory droplet nuclei as a function of time for various heights of the vibrating mesh nebulizer. For a height of 55 cm, which is similar to the height of the air purifier intake (54 cm), the simulated respiratory droplet nuclei maintained a lower concentration compared to when the air purifier was not operating. This observation showed that most of the simulated respiratory droplet nuclei were removed by the air purifier. However, with the vibrating mesh nebulizer at heights of 70 cm and 100 cm, the spread of the simulated respiratory droplet nuclei was observed during the early stage of air purifier operation. This observation shows that the simulated respiratory droplet nuclei can be transmitted with the airflow generated by exhaust from the air purifier, as reported previously by Ham [10]. With the vibrating mesh nebulizer at a height > 70 cm, the difference in the concentration was negligible. These observations indicated that the airflow generated by the air purifier can spread respiratory droplet nuclei when the source is close to the exhaust of the air purifier. However, in comparison with the number concentration without the air purifier, the number concentration was lower after 2 min of operation of the air purifier, even with the source located near the exhaust. Without the air purifier, the simulated respiratory droplet nuclei accumulated in the chamber and, therefore, the concentration continued to increase. However, continuous operation of the air purifier removed simulated respiratory droplet nuclei, because the air was recirculated and the air purifier contained an H13 HEPA filter with a removal efficiency of 99.99% for particles in the size range of the respiratory droplet nuclei [12,13]. Therefore, with the exception of the early stage of air purifier operation, the removal effect of the air purifier was dominant in relation to the spread effect, even with the source close to the exhaust. We also measured the concentration of the simulated respiratory droplet nuclei for various distances between the nebulizer and the air purifier to evaluate the characteristics of the spread of simulated respiratory droplet nuclei by the air purifier. In this experiment, the height of the vibrating mesh nebulizer was fixed at 70 cm. Figure 2b shows the changes in the concentration of the simulated respiratory droplet nuclei with time. The variation in the concentration of simulated respiratory droplet nuclei with distance between the air purifier and source was not significant. Consequently, the simulated respiratory droplet nuclei were not significantly affected by the airflow generated by the air purifier. The results showed that operation of the air purifier is beneficial to maintain a low concentration of respiratory droplet nuclei, i.e., to reduce the probability of infection [14,15].

Under conditions of low humidity, the moisture of respiratory droplet nuclei can be completely evaporated, and nano-sized single virus particles or aggregates of several viruses can be formed [16]. The inhaled dose of a single virus or aggregates of viruses is very low and, therefore, the probability of infection by these nano-sized aggregates may be low [17]. However, the nano-sized particles can be suspended in the air for more than 12 h and, therefore, virus aggregates can accumulate in the air [18]. Previous experiments showed that simulated respiratory droplet nuclei can also be evaporated due to airflow, and nano-sized aggregates of PSL particles can be generated.

**Figure 3:**
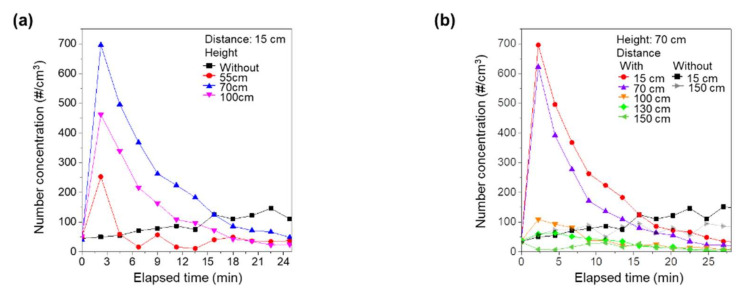
Change in the concentration of nano-sized aggregates as a function of time (**a**) for various heights of source and (**b**) for various distances between the air purifier and the source.

To determine the effects of the air purifier on nano-sized virus aggregates, we replaced APS with SMPS. We repeated the experiments and measured the concentration of nano-sized aggregates. Figure 3a shows the measured concentration of nano-sized aggregates with time for various heights of the source and distances between the air purifier and the source. Due to their high mobility, the spread of nano-sized aggregates by the air purifier was markedly increased in comparison to the simulated respiratory droplet nuclei. Even with the source located near the intake of the air purifier, i.e., a source height of 55 cm, the concentration with air purifier operation was higher than that without air purifier operation at the early stage due to the high diffusivity of nano-sized particles [19]. However, after 5 min of operation of the air purifier, the removal effect was dominant, as shown in Figure 3a. With the source located near the exhaust of the air purifier, i.e., source heights of 70 cm and 100 cm, the spread of nanoaggregates was much higher. The concentration remained higher than that without operation of the air purifier up to 15 min. Therefore, the spreading of the nano-sized aggregates by the operation of the air purifier was much higher than for the simulated respiratory droplet nuclei.

We also examined the effects of distance between the source and the air purifier on the spread of nano-sized aggregates. With a constant source height of 70 cm, we changed the distance between the air purifier and the source and measured the concentration of nano-sized aggregates, as shown in Figure 3b. When the source was located near the air purifier, the air purifier vigorously spread the nano-sized aggregates. Interestingly, however, the spread of nano-sized aggregates by the operation of the air purifier was markedly suppressed at distances > 100 cm. For distances of 130 cm and 150 cm, the number concentration of nano-sized aggregates was always lower than without operation of the air purifier. With sufficient distance from the air purifier, the nano-sized aggregates generated by the vibrating mesh nebulizer were not directly affected by the exhaust airflow, and, under these conditions, operation of the air purifier would help to reduce infection.

## 4. Conclusions

In this study, we evaluated the spreading characteristics of simulated respiratory droplet nuclei and nano-sized aggregates due to the operation of an air purifier. The results showed that the effect of the air purifier on the spread of micron-sized respiratory droplet nuclei was not significant, and the operation of the air purifier was beneficial for reducing the concentration of infectious respiratory droplet nuclei. However, the nano-sized aggregates, which can represent a single virus or aggregates of viruses, were significantly affected by the airflow generated by the air purifier. A single virus or an aggregate of viruses has a low inhalation dose, but can accumulate and remain in the air for more than 12 h. The airflow generated by the air purifier vigorously spread nano-sized aggregates, particularly at the early stage of operation of the air purifier. However, removal of the nano-sized aggregates by the air purifier was dominant, because filtered air was continuously supplied by the air purifier. In addition, when the source of the nano-size aggregates was more than 1 m from the air purifier, the spreading effect was markedly reduced. Therefore, for both respiratory droplet nuclei and aggregates of viruses, the operation of the air purifier can help to reduce the concentration, and thus reduce the airborne transmission rate.

Some limitations of the present study should be acknowledged. The results were obtained under the specific conditions adopted in the present study, i.e., no ventilation was available, the test chamber had a fixed volume, and the air purifier had a set flow rate. Further experimental and computational studies on the spread of viruses, including the combined effects of ventilation and an air purifier, as well as the relation between the clean air discharge rate (CADR) of the air purifier and the volume of the indoor facility, are required to develop guidelines for the safe usage of air purifiers.
